# A longitudinal analysis of the role of potentially morally injurious events on COVID-19-related psychosocial functioning among healthcare providers

**DOI:** 10.1371/journal.pone.0260033

**Published:** 2021-11-12

**Authors:** Lauren M. Borges, Ryan Holliday, Sean M. Barnes, Nazanin H. Bahraini, Adam Kinney, Jeri E. Forster, Lisa A. Brenner

**Affiliations:** 1 Rocky Mountain Mental Illness Research, Education and Clinical Center for Veteran Suicide Prevention, Aurora, Colorado, United States of America; 2 Department of Psychiatry, University of Colorado Anschutz Medical Campus, Aurora, Colorado, United States of America; 3 Department of Physical Medicine and Rehabilitation, University of Colorado Anschutz Medical Campus, Aurora, Colorado, United States of America; Uniformed Services University of the Health Sciences, UNITED STATES

## Abstract

Medical leaders have warned of the potential public health burden of a “parallel pandemic” faced by healthcare workers during the COVID-19 pandemic. These individuals may have experienced scenarios in which their moral code was violated resulting in potentially morally injurious events (PMIEs). In the present study, hierarchical linear modeling was utilized to examine the role of PMIEs on COVID-19 pandemic-related difficulties in psychosocial functioning among 211 healthcare providers (83% female, 89% White, and an average of 11.30 years in their healthcare profession [9.31]) over a 10-month span (May 2020 –March 2021). Reported exposure to PMIEs was associated with statistically significant poorer self-reported psychosocial functioning at baseline and over the course of 10-months of data collection. Within exploratory examinations of PMIE type, perceptions of transgressions by self or others (e.g., “I acted in ways that violated my own moral code or values”), but not perceived betrayal (e.g., “I feel betrayed by leaders who I once trusted”), was associated with poorer COVID-19 related psychosocial functioning (e.g., feeling connected to others, relationship with spouse or partner). Findings from this study speak to the importance of investing in intervention and prevention efforts to mitigate the consequences of exposure to PMIEs among healthcare providers. Interventions for healthcare providers targeting psychosocial functioning in the context of moral injury is an important area for future research.

## Introduction

It has been suggested that immediate action is required to prevent a “parallel pandemic” associated with healthcare providers’ exposure to potentially morally injurious events (PMIEs) during the coronavirus disease 2019 (COVID-19) pandemic, and the mental health consequences that may follow [1]. PMIEs can be defined as situations in which one’s moral code was violated either through their own transgressive actions or inactions, other peoples’ transgressive actions or inactions, or through perceived betrayal by others [2]. In conceptual papers about exposure to PMIEs among providers, the ways in which healthcare workers may have experienced violations of their moral code during the pandemic is described, along with the potential for moral injury [3–7]. PMIEs among providers may have included a number of high-stakes situations associated with providing care during the pandemic including triaging or discharging COVID-19 patients amidst a lack of resources (e.g., when resources are limited choosing which patient receives a ventilator, negative pressure room, or oxygen first), working directly with COVID-19 patients dying alone without family members (e.g., a nurse providing care who must tell a dying patient that their family cannot visit), witnessing others engaging in unethical practices related to COVID-19 and not intervening (e.g., watching a provider inappropriately prioritize care for a patient without a chronic medical condition, delaying care for others and not intervening), following orders by hospital administrators to engage in unethical practices related to COVID-19 (e.g., avoiding care practices due to personal safety concerns, causing the patient harm by delaying their care), and/or other similar events [3,4]. In one study where the prevalence of physician exposure to PMIEs was assessed in the context of COVID-19, almost 50% of the physicians surveyed reported exposure to PMIEs [8]. In a qualitative investigation, moral distress and moral injury were found to be the primary stressors experienced by healthcare workers providing care during the COVID-19 pandemic [9]. Finally, in a different qualitative study, when asked about stressors during the pandemic, nurses’ responses were consistent with those described among individuals experiencing moral injury [10].

However, little is known about the consequences of exposure to PMIEs for healthcare providers and how these consequences might affect patients and healthcare systems, as the preponderance of research on exposure to PMIEs concerns Active-Duty Service Members and warzone Veterans. In conceptual papers about moral injury in healthcare providers during the pandemic, one potential consequence of exposure to PMIEs that has been emphasized is a relationship between PMIEs and decreased psychosocial functioning. Moreover, some models of moral injury highlight that moral injury occurs among healthcare providers when psychosocial functioning is impacted as a result of exposure to PMIEs [3,4,7,11]. Because exposure to PMIEs tends to involve a departure from one’s social values, a central element of moral injury often includes difficulties in psychosocial functioning associated with meaningfully engaging in interpersonal relationships. For example, if interpersonal contexts evoke contact with one’s moral code violations (e.g., interacting with my child is a reminder I didn’t save the life of a young child’s parent who died of COVID-19), it is likely that the scenarios eliciting these painful experiences will be avoided. Such avoidance precludes engagement in meaningful life experiences (e.g., avoiding spending time with my child). Moreover, when individuals avoid engaging in important areas of their life, they are often at risk for mental health consequences [12].

While research is still limited, the mental health consequences of healthcare providers’ exposure to PMIEs and moral distress in the context of the COVID-19 pandemic are beginning to be investigated. Several cross-sectional studies with samples of individuals from different countries (China, Spain, and the United States [US]), have shown a relationship between exposure to PMIEs and greater risk for anxiety, depression, posttraumatic stress disorder (PTSD), burnout, sleep difficulties, and suicidal ideation and self-directed violence [13–15].

In one US study, follow-up data were collected at one and three months indicating that healthcare providers working in less supportive environments endorsed higher levels of exposure to PMIEs [16]. Although important in building a foundation for investigating moral injury among healthcare providers, these studies include several limitations, most notably small sample size, lack of long-term follow-up, limited statistical analyses, conflation of exposure to PMIEs with impact of moral injury, and lack of measurement of psychosocial functioning related to COVID-19.

The present study is the first to explore the longitudinal relationship between exposure to PMIEs associated with the COVID-19 pandemic and psychosocial functioning among healthcare workers. Both PMIEs and psychosocial functioning relative to COVID-19 were measured across 10-months using self-report measures to better understand the extent to which exposure to PMIEs influenced psychosocial functioning among providers (e.g., relationships, recreation, spirituality, work). We hypothesized that providers reporting exposure to PMIEs would exhibit significantly poorer psychosocial functioning relative to those not reporting experiencing a PMIE across 10-months.

## Method

### Participants & procedure

Recruitment and study procedures were approved by the Colorado Multiple Institutional Review Board and VA Research and Development committees. Participants were eligible if they 1) were a healthcare provider who delivered outpatient and/or inpatient treatment during the COVID-19 pandemic, 2) delivered health care services in the United States, and, 3) delivered services during the pandemic (on or after January 20, 2020). Participants were recruited by leveraging professional networks of study personnel, displaying flyers in healthcare settings, and disseminating study information on social media platforms, which read, “we are seeking health care providers to participate in a research study about the impact of COVID-19 on their physical and mental health.” Snowball sampling methods were used to recruit additional participants. A HIPAA-compliant online survey platform was utilized to document informed consent and collect self-report data. During the baseline survey, providers completed a measure of demographic characteristics, PMIE exposure, and psychosocial functioning. They were then prompted to complete follow-up online surveys every four weeks for one year to collect data on PMIE exposure and psychosocial functioning. Although 12 months of data were collected, 10 months of longitudinal data are reported to avoid unreliable parameter estimates secondary to small sample sizes at months 11 and 12. In total, 211 healthcare providers were recruited from May 2020 to March 2021. The majority of participants were women (82.76%), White (88.73%), and mental health professionals (61.14%). Please see Table 1 for additional sociodemographic and occupation-related characteristics.

**Table 1:** Sociodemographic and work-related sample characteristics (N = 211).

Variable	*M*	*SD*
Years in profession	11.80	9.31
	*n*	%
Sex		
Male	33	16.26
Female	168	82.76
Other	2	.99
Race		
White	181	89.16
Non-White or multiracial	22	10.84
Profession		
Physician	26	12.32
Nurse	22	10.43
Mental health provider	129	61.14
Physical therapist	8	3.79
Occupational therapist	4	1.90
Nurse practitioner	5	2.37
Physician assistant	1	.47
Technician	1	.47
EMT/Paramedic	1	.57
Other	14	6.64

*Note*. Data were missing as follows: sex (*n* = 8) and race (*n* = 8).

### Measures

A modified version of the 9-item Moral Injury Events Scale (MIES) was administered to assess exposure to PMIEs among healthcare workers in the context of providing health care during the COVID-19 pandemic [2]. This measure allowed participants to select responses on a 6-point Likert-type scale including the options 1 “strongly agree,” 2 “moderately agree,” 3 “slightly agree,” 4 “slightly disagree,” 5 “moderately agree,” and 6 “strongly disagree.” Given this range, a lower score is associated with greater exposure to PMIEs. Wording was revised in the instructions so that experiences “… providing health care during the COVID-19 pandemic” could be assessed rather than “experiences on deployment.” Additionally, items 8 and 9 of the MIES were modified to make them specific to healthcare providers, changing “service members” to “providers” in item 8 and “military” to “medical field” in item 9. The modified MIES is a self-report measure which was used to screen for exposure to PMIEs as well as specific types of PMIEs (i.e., perceived transgressions by self or others; perceived betrayal by others). For the purpose of the current analysis, MIES scores were dichotomized such that a response of “Moderately Agree” to “Strongly Agree” on any of the 9 MIES items was coded as an exposure to a PMIE having occurred. Additionally, if the provider reported an affirmative response of “Moderately Agree” to “Strongly Agree” on items 1–6 (i.e., perceived transgressions by self or others; e.g., “I saw things that were morally wrong,” “I acted in ways that violated my own moral code or values,” “I violated my morals by failing to do something I felt I should have done,”) and items 7–9 (i.e., perceived betrayal by others; e.g., “I feel betrayed by leaders who I once trusted,” “I feel betrayed by fellow medical providers who I once trusted,”) then these specific PMIEs were coded as having occurred. This dichotomous scoring approach has been used in several prior investigations [17,18]. Additionally, the MIES has been found to have strong internal validity, temporal stability, and concurrent validity [2].

At the start of the COVID-19 pandemic, there were no extant measures suitable for assessment of COVID-19 related difficulties in psychosocial functioning. Hoffmire and colleagues (under review), as part of a larger surveillance effort of non-fatal suicidal self-directed violence, created a face-valid 18-item measure of the negative impact of the COVID-19 pandemic on psychosocial functioning, the Perceived Impact of Pandemic Scale-18 (PIPS-18) [19]. The structure and scoring of the scale were modeled after other measures of trauma-related psychosocial functioning deficits (e.g., Brief Inventory of Psychosocial Functioning; B-IPF) [20]. Participants were asked to rate “how much each of the following areas of your life has been negatively impacted by the COVID-19 pandemic” on a 5-point scale of “not at all,” “a little,” “somewhat,” “a lot,” and “very much” in addition to the option to respond as “not applicable.” Participants were asked to rate their response associated with the following categories of psychosocial functioning: relationship with spouse or partner, parenting, ability to care for family, friendships, work (loss of hours, loss of job, ability to attain employment, productivity), finances, retirement, education or training, ability to engage in recreational activities, spirituality or religiosity, ability to engage in household chores, mental health, ability to obtain needed health care, and feeling connected to others. Total scores were calculated by summing scale items completed by respondents (not including items marked as “not applicable”) and dividing by the maximum possible score based on applicable items. This value was then multiplied by 100, with higher scores being indicative of more difficulties with functioning.

Additional measures were included as part of the parent study. Only measures relevant to the current study hypotheses are reported.

### Analytic approach

To ensure groups did not differ based on demographic and job-related characteristics, between-group comparisons were conducted based on reported exposure to PMIE(s). Three analyses were conducted for each demographic/job-related characteristic, including any reported exposure to a PMIE in general, reported exposure to a PMIE through transgression by self/other, and reported exposure to a PMIE through perceived betrayal. An independent samples *t*-test was utilized to examine differences based on years in profession. Chi-square or Fisher’s exact analyses, as appropriate, were conducted to examine differences based on sex, race, and profession. Given cell sizes, only those reporting male or female sex were included in sex analyses. Additionally, we elected to collapse several cells for profession to facilitate analyses. We grouped medical providers (i.e., physician, nurse, nurse practitioner, physician assistant, phlebotomist, EMT/paramedic, technician) as well as physical and occupational therapists. Factors found to significantly differ at baseline would have been included as covariates in subsequent models.

Hierarchical linear modeling was conducted using HLM version 8. This approach was chosen to efficiently handle unbalanced designs and missing longitudinal data, allowing for observations to vary more effectively than other longitudinal analytic methods (e.g., repeated measure analysis of variance) [21,22].

Level-1 data (within-person) included repeated measures of psychosocial functioning for the 10-month period. Data were centered at baseline to assess for initial differences in functioning between those who did and did not endorse experiencing PMIE(s). Model fit was examined for each model by assessing deviance and conducting a chi-square difference test between models. Linear, quadratic, piecewise, and logarithmic growth curves were specified, with a logarithmic growth curve having the best model fit [23,24].

Prior to nesting Level-1 data within upper level (i.e., Level-2; between-person based on PMIE exposure) units, we confirmed that the unconditional growth curve was significant and thus appropriate for the inclusion of a Level 2 predictor [23]. Based on the significance of the unconditional growth curve, PMIE exposure (dichotomous: yes/no) was coded and included as a Level-2 predictor based on the first time point the healthcare provider reported the exposure occurred. For example, if a provider reported a PMIE having occurred 3-months into the study, they were coded as having PMIE exposure at 3 months onward, but not for the prior 2 months. PMIEs were only measured related to providing care during the COVID-19 pandemic.

Exposure to reported PMIEs was utilized to assess the primary hypothesis for the current analysis (see S1 Table for rates of reported exposure to PMIEs). Exploratory follow-up analyses were conducted based on exposure to perceived transgressions by self or others, as well as perceived betrayal by others. A standard level of significance (*p* < .05) was utilized for all models.

## Results

At baseline, no demographic or job-related characteristics were found to significantly differ based on report of PMIE (*p* > .05; see S2 Table). Similarly, none of these characteristics were significantly different based on report of transgression by self/other or perceived betrayal (*p* > .05; see S3 and S4 Tables). As such, no factors were included as covariates in subsequent models.

Results of the unconditional growth curve indicated a significant logarithmic slope of change in psychosocial functioning over the course of the 10 months (*p* < .05). In particular, on average, providers experienced initial increases in functioning which stabilized over the course of the 10 months.

**Fig 1:**
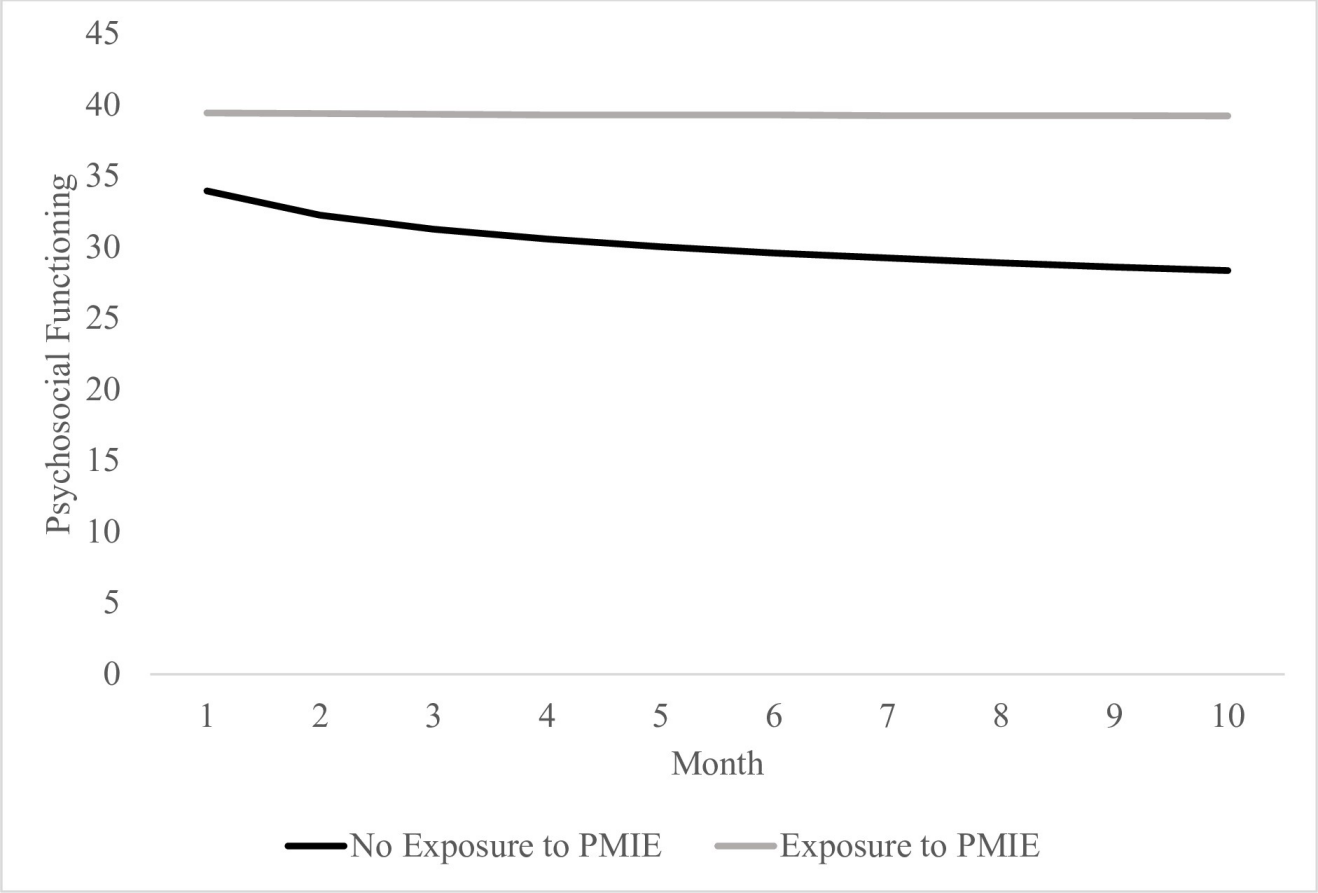
Logarithmic change in psychosocial functioning over the course of 10 months during the COVID-19 pandemic based on PMIE exposure.

When PMIE was added as a Level-2 variable, across providers, PMIE was associated with logarithmic change in psychosocial functioning (see Table 2 and Fig 1). In particular, reported exposure to a PMIE was significantly related to changes in psychosocial functioning over the course of the 10-month study period, *t*(243, 1.00) = 2.33, *b* = 2.34, *p* = .020. Specifically, those who did not report experiencing a PMIE appeared to demonstrate initial improvements in psychosocial functioning which stabilized over time; however, those reporting experiencing a PMIE appeared to experience relatively minimal improvement in functioning over the course of the study period. The main effect of PMIE on the intercept was similarly significant, *t*(243, 1.92) = 2.86, *b* = 5.48, *p* = .005, suggesting that those noting PMIE exposure at baseline had poorer initial functioning.

**Table 2:** Hierarchical linear model of the role of PMIE exposure on psychosocial functioning over the course of 10 months among healthcare providers during the COVID-19 pandemic.

Fixed effect	*b*	*SE*	*t*	*p*
Intercept	33.97	1.23	27.65	< .001
PMIE exposure	5.48	1.92	2.86	.005
Slope				
Intercept	-2.43	.64	-3.78	< .001
PMIE exposure	2.34	1.00	2.33	.02
Random effect	Variance		χ^2^	*p*
Intercept	187.42		888.21	< .001
Slope	19.63		345.12	< .001
Level-1 *r*	43.71			

*Note*. PMIE = potentially morally injurious event. PMIE exposure coded as reported exposure (= 1) vs. did not report exposure (= 0).

**Fig 2:**
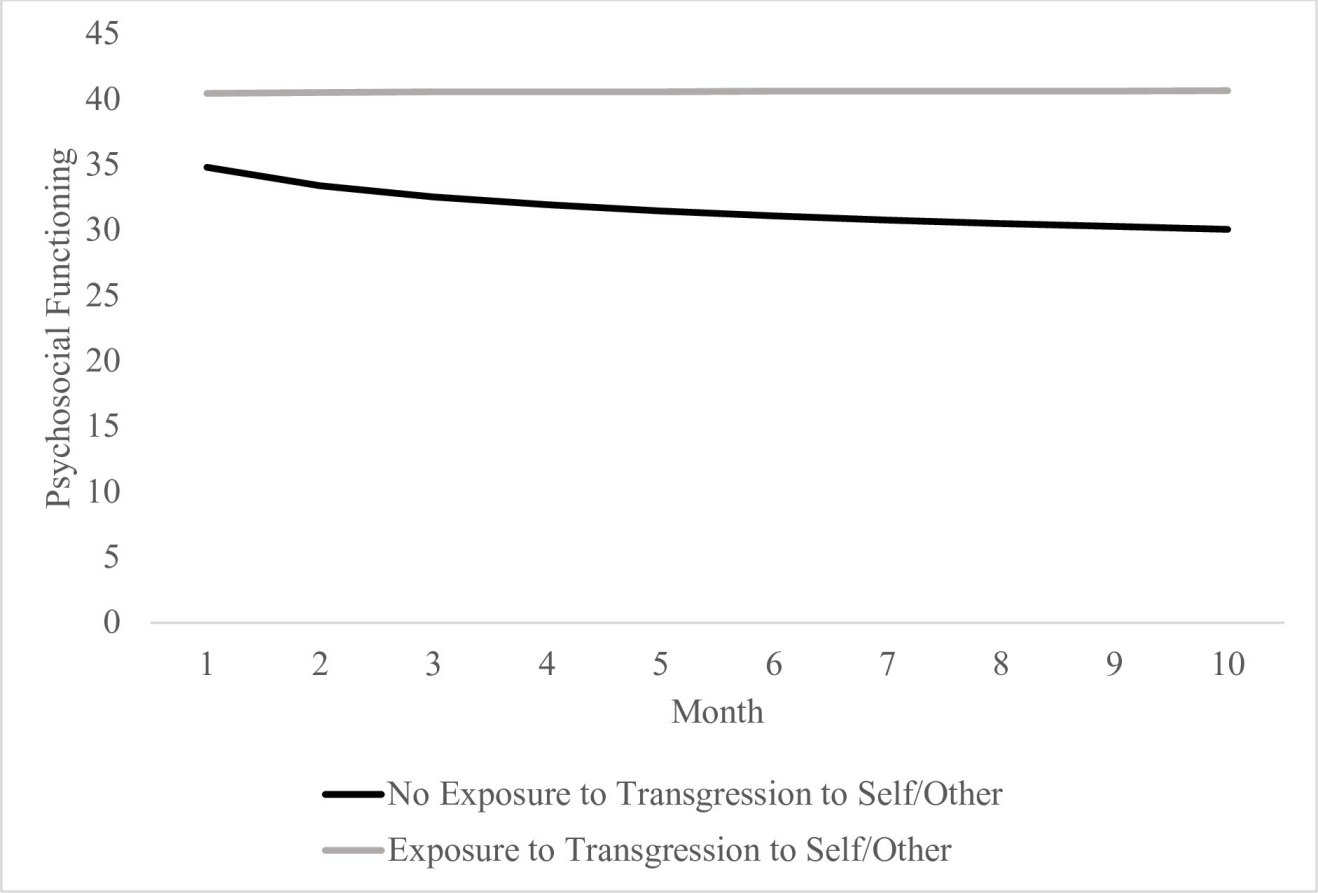
Logarithmic change in psychosocial functioning over the course of 10 months during the COVID-19 pandemic based on transgression to self or other exposure.

**Fig 3:**
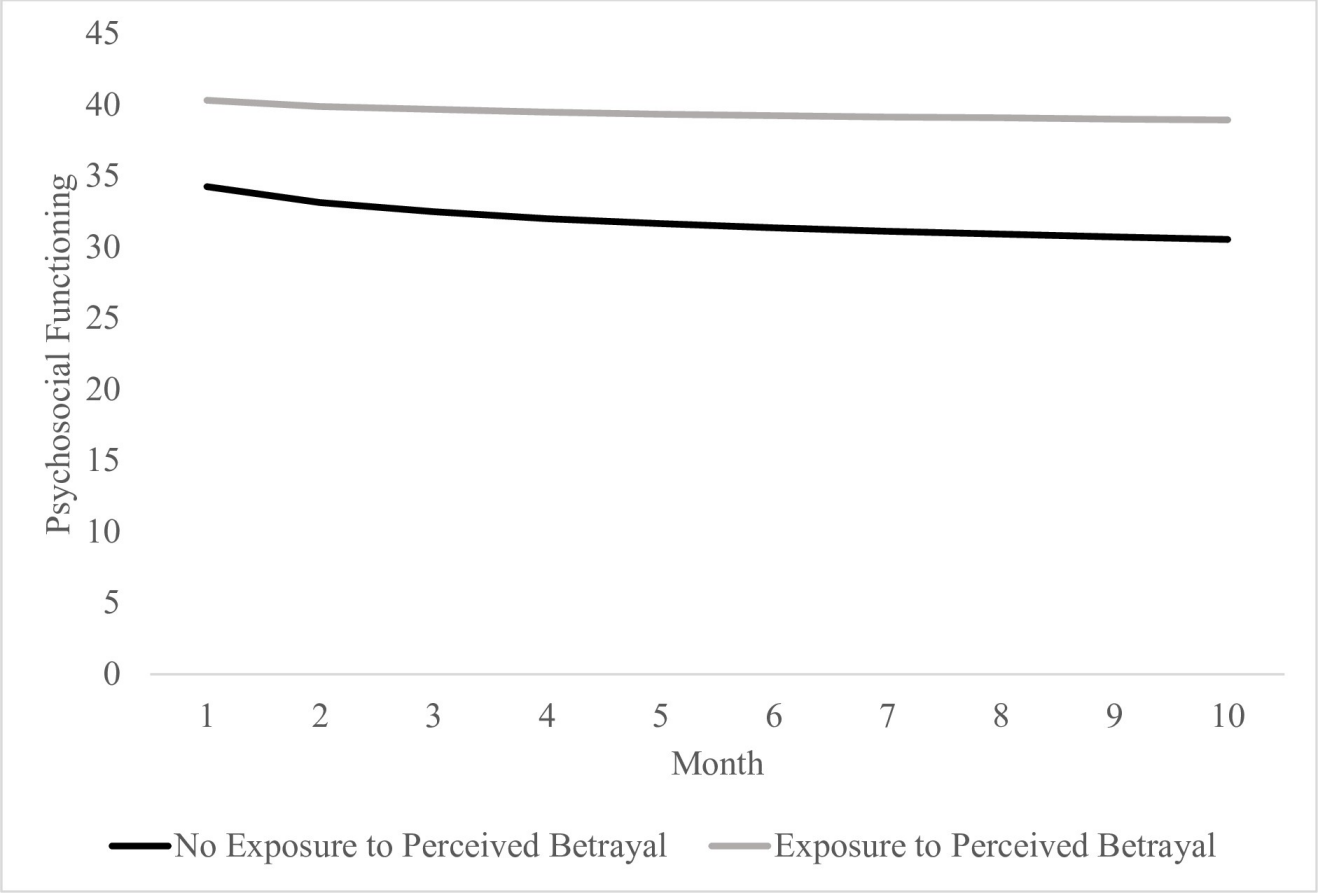
Logarithmic change in psychosocial functioning over the course of 10 months during the COVID-19 pandemic based on perceived betrayal.

Additional exploratory analyses based on exposure to transgressions by self or others, as well as perceived betrayal by others, were also conducted (see Tables 3 and 4 and Figs 2 and 3). Results show that those reporting exposure to transgressions by self or other appeared to experience minimal change in psychosocial functioning relative to logarithmic improvements in functioning experienced by those who did not report exposure to transgressions by self or others over the 10-month study period, *t*(243, 1.07) = 2.02, *b* = 2.15, *p* = .045. Report of perceived betrayal was not found to be significantly associated with logarithmic slope, *t*(243, 1.08) = 0.93, *b* = 1.01, *p* = .345. As such, both those who did and did not report perceived betrayal appeared to experience similar improvements in psychosocial functioning over 10 months. Additionally, the main effect of transgressions by self or others, *t*(243, 2.13) = 2.65, *b* = 5.64, *p* = .009, and perceived betrayal, *t*(243, 2.03) = 2.98, *b* = 60.7, *p* = .003, were significant at intercept, suggesting that those reporting these experiences appeared to have poorer baseline psychosocial functioning relative to those not reporting these experiences.

**Table 3:** Hierarchical linear model of the role of perceived transgression to self or other exposure on psychosocial functioning over the course of 10 months among healthcare providers during the COVID-19 pandemic.

Fixed effect	*b*	*SE*	*t*	*p*
Intercept	34.81	1.11	31.41	< .001
PMIE exposure	5.65	2.13	2.65	.009
Slope				
Intercept	-2.06	.62	-3.30	.001
PMIE exposure	2.15	1.07	2.02	.045
Random effect	Variance		χ^2^	*p*
Intercept	187.78		888.44	< .001
Slope	20.36		352.05	< .001
Level-1 *r*	43.56			

*Note*. Transgression to self or other coded as reported exposure (= 1) vs. did not report exposure (= 0).

**Table 4:** Hierarchical linear model of the role of perceived betrayal on psychosocial functioning over the course of 10 months among healthcare providers during the COVID-19 pandemic.

Fixed effect	*b*	*SE*	*t*	*p*
Intercept	34.27	1.13	30.46	< .001
PMIE exposure	6.07	2.03	2.98	.003
Slope				
Intercept	-1.61	.62	-2.60	.010
PMIE exposure	1.00	1.08	.93	.354
Random effect	Variance		χ^2^	*p*
Intercept	186.17		908.21	< .001
Slope	21.13		359.76	< .001
Level-1 *r*	43.55			

*Note*. Perceived betrayal coded as reported exposure (= 1) vs. did not report exposure (= 0).

## Discussion

Among healthcare providers included in this study experiencing a PMIE at baseline was associated with poorer psychosocial functioning relative to no PMIE exposure. Additionally, those reporting exposure to a PMIE appeared to experience no relative improvement in functioning over 10-months (see Fig 1). Conversely, those reporting no exposure to a PMIE appeared to improve in functioning over time. Exposure to PMIEs therefore appears to be a potential driver of longitudinal trajectories of psychosocial functioning. The current study is consistent with the idea that a “parallel pandemic” related to the mental health burden of providing care during the COVID-19 pandemic is a relevant public health concern for the millions of healthcare workers providing care during the pandemic [1,25].

Related to exposure to specific kinds of PMIEs, self and other directed PMIEs were associated with decreased improvement in functioning over time while betrayal related PMIEs were not (see Figs 2 and 3). Research in Veterans similarly suggests that betrayal-related PMIEs are associated with worse functioning at baseline, but that perpetration related/other directed PMIEs most reliably predict assignment to trajectories characterizing poor or declining functioning [26]. It may be that individuals who do something to violate their moral code (e.g., directly or by failing to stop others from engaging in immoral acts) have more difficulty reengaging their values after this violation, leading to poorer functioning, than those who are betrayed.

The findings from this study are relevant in beginning to understand the impact of provider exposure to PMIEs on the healthcare industry as jobs associated with healthcare employ a larger number of Americans than any other industry in the United States, accounting for 20,498,753 workers in 2018 [27]. Investigating the influence of PMIEs on healthcare provider functioning during this global health emergency is an important step in understanding how to invest in the most efficient and effective prevention and intervention efforts for this large and vital population. The consequences of exposure to PMIEs could inhibit providers’ ability to sustain delivery of critical care during a global public health crisis. To better understand how to protect and invest in healthcare workers, longitudinal studies like this one can help increase understanding of the impact of PMIEs on healthcare providers’ functioning in the context of the COVID-19 pandemic.

In finding that PMIEs are associated with poorer functional improvement over time, it is important to consider resources for intervention and prevention to protect healthcare providers, patients, and the infrastructure of healthcare systems. Engaging interventional strategies that target functional outcomes (e.g., relationship engagement, connection to work, engagement with spirituality, practice of self-care) while facilitating flexibility in responding to the painful experiences PMIEs can evoke (e.g., guilt, shame, anger, disgust, and contempt) may be particularly prudent. For instance, if an individual is demonstrating poor psychosocial functioning because they are no longer going to work following the experience of a PMIE, this could be because participating in work puts them into direct contact with their experience of violating their moral code (e.g., memories, emotions, and thoughts associated with this experience). If it is conceptualized that avoidance of these painful experiences prevents the provider from engaging meaningfully in their life (e.g., this individual previously ascribed tremendous meaning to their role as a healthcare provider), helping this individual reconnect with work while experiencing painful thoughts and emotions that arise in the presence of their role as a healthcare provider could be critical to their recovery. A number of interventions focused on cognitive, behavioral, and social processes have been applied to the treatment of moral injury in warzone Veterans and Service-Members which may be relevant to helping healthcare providers respond to moral distress more flexibly [28–30]. Interventions, such as Acceptance and Commitment Therapy, which have been developed to target both psychosocial functioning in moral injury [27,28] and psychosocial functioning within healthcare providers [31,32], may be particularly beneficial.

Additionally, investing in prevention efforts to mitigate exposure to PMIEs could be an important approach for addressing the public health burden of exposure to PMIEs and preventing the development of moral injury among healthcare providers. Interventions that target change at levels beyond the individual provider may be critical in preventing moral injury. Specifically, some experts have proposed changes at the level of healthcare organizations [1,4]. These changes could include implementing triage committees so that the burden of high stakes decisions related to patient care during a pandemic is spread across groups of providers rather than falling onto individual providers, creating anonymous reporting mechanisms for healthcare providers to use to express concerns about hospital policy and patient care, and creating a culture where employee wellness is factored into workday scheduling and productivity [1,4,5].

The World Health Organization has designated 2021 the year of health and care workers [33], calling for protection and investment in healthcare workers. Changes at the level of national policy have been proposed, including the allocation of funding to measure clinician wellbeing and treating the physical and mental health consequences associated with providing care during the COVID-19 pandemic [1]. In fact, a bill named for an Emergency Room doctor who died by suicide at the peak of the COVID-19 pandemic, the Dr. Lorna Breen Health Care Provider Protection Act [34], has recently been proposed to support healthcare workers’ mental health in the context of the COVID-19. Efforts like these will be critical to facilitating prevention of moral injury and improved mental health functioning in providers who have worked in overtaxed healthcare systems during the COVID-19 pandemic.

While the current study represents a robust longitudinal examination of the role of PMIEs on functioning among healthcare workers providing care during the COVID-19 pandemic, some limitations are of note. The sample may not generalize to the larger healthcare provider workforce due to sample size and characteristics, namely that the majority of providers were white, female, and also mental health workers. While no differences were noted at baseline between PMIEs and profession, job-related differences may be relevant to trajectories of functioning in the context of moral injury. Such differences may be especially notable within professions with greater potential exposure to patients with COVID (e.g., those working with emergency department settings or intensive care units). As such, additional research based on functioning, profession, and moral injury remains warranted.

Additionally, while HLM analyses are equipped to handle missing data, attrition was still notable in the sample. We opted to use measures with sensitivity to the specific stressors of COVID-19 in the current study so that we could better understand the potential impact of PMIEs on psychosocial functioning. Therefore, the MIES was slightly modified to make it relevant to healthcare providers during COVID-19, thus the psychometric properties of the scale with these modifications are unknown. A new measure of psychosocial functioning associated with COVID-19, the PIPS-18, was developed. While this measure was informed by other measures of trauma-related psychosocial functioning deficits (e.g., B-IPF), the psychometric properties of the scale are unknown.

Another limitation of the study design is that we cannot specifically determine whether PMIEs drove changes in functioning or if these changes are attributed to other factors. We also examined overall impact on functioning, rather than specific domains. Therefore, additional research on types of functional domains affected by pandemic-related PMIEs is likely warranted. Additionally, PMIEs were examined dichotomously, rather than continuously, limiting the ability to infer the impact of exposure to multiple PMIEs. Finally, given our sampling method, bias associated with those who responded to the survey is also a limitation.

The results of this study are compelling and support the importance of addressing psychosocial functioning and exposure to PMIEs among healthcare workers providing care in the context of the pandemic. Future research explicitly exploring the development of moral injury among healthcare providers is an important next step. In particular, better understanding the pathways through which moral injury emerges in healthcare providers is important to tailoring treatment efforts. For instance, investigating how healthcare providers respond to distressing moral emotions (guilt, shame, contempt, anger, disgust) and cognitions (self-blame thoughts [e.g., “It’s my fault the patient died”]; other-blaming thoughts [e.g., “My supervisor is evil”]) and the extent to which this pattern of responding gives rise to difficulties in psychosocial functioning, particularly in domains connected to moral injury in the literature like social relationships and self-care is a critical area of future research.

More research is also needed to investigate how provider exposure to PMIEs affects patient care and healthcare systems. To truly comprehend the public health implications of exposure to PMIEs, the impact of provider moral injury needs to be investigated in the context of employee turnover and patient safety. Such studies may help to inform public policy changes (e.g., resources to support provider teams in identifying and acting consistently with shared values) and occupational health measures (e.g., implementation of evidence-based interventions for healthcare providers struggling related to moral injury). These efforts could prevent negative outcomes associated with moral injury at the level of individual healthcare workers (e.g., suicidal behavior as a result of the consequences of exposure to PMIEs) and at the level of systems of care (e.g., loss of essential workforce, loss of patient access to critical care).

## Supporting information

S1 Table Rates of reported exposure to PMIE at each month. (DOCX)

S2 Table Sociodemographic and work-related sample characteristics based on reported exposure to a PMIE. (DOCX)

S3 Table Sociodemographic and work-related sample characteristics based on reported exposure to transgression to self/other. (DOCX)

S4 Table Sociodemographic and work-related sample characteristics based on reported exposure to perceived betrayal. (DOCX)
