# Supplementary material for: A longitudinal analysis of the role of potentially morally injurious events on COVID-19-related psychosocial functioning among healthcare providers
Source: PLoS One. 2021 Nov 12;16(11):e0260033. doi: 10.1371/journal.pone.0260033 (PMC8589198; doi:10.1371/journal.pone.0260033)
Supplement: S1 Table — (DOCX) [file pone.0260033.s001.docx]

S1 Table.

*Rates of Reported Exposure to PMIE at Each Month*

| Month | Reported exposure | | No reported exposure | |
| --- | --- | --- | --- | --- |
|  | *n* | % | *n* | % |
| 1 |  |  |  |  |
| Any PMIE Exposure | 109 | 51.66 | 102 | 48.34 |
| Transgression by self/other | 77 | 36.49 | 134 | 54.71 |
| Perceived betrayal | 88 | 41.71 | 123 | 50.29 |
| 2 |  |  |  |  |
| Any PMIE Exposure | 53 | 45.69 | 63 | 54.31 |
| Transgression by self/other | 33 | 28.45 | 83 | 71.55 |
| Perceived betrayal | 47 | 40.52 | 69 | 59.48 |
| 3 |  |  |  |  |
| Any PMIE Exposure | 50 | 47.17 | 56 | 52.83 |
| Transgression by self/other | 28 | 26.42 | 78 | 73.58 |
| Perceived betrayal | 46 | 43.40 | 60 | 56.60 |
| 4 |  |  |  |  |
| Any PMIE Exposure | 43 | 46.74 | 49 | 53.26 |
| Transgression by self/other | 22 | 23.91 | 70 | 76.09 |
| Perceived betrayal | 42 | 45.65 | 50 | 54.35 |
| 5 |  |  |  |  |
| Any PMIE Exposure | 46 | 52.27 | 42 | 47.73 |
| Transgression by self/other | 28 | 31.82 | 60 | 68.18 |
| Perceived betrayal | 39 | 44.32 | 49 | 55.68 |
| 6 |  |  |  |  |
| Any PMIE Exposure | 43 | 54.43 | 36 | 45.57 |
| Transgression by self/other | 23 | 29.11 | 56 | 70.89 |
| Perceived betrayal | 43 | 54.43 | 36 | 45.57 |
| 7 |  |  |  |  |
| Any PMIE Exposure | 35 | 50.72 | 34 | 49.28 |
| Transgression by self/other | 22 | 31.88 | 47 | 68.12 |
| Perceived betrayal | 32 | 46.38 | 37 | 53.62 |
| 8 |  |  |  |  |
| Any PMIE Exposure | 31 | 43.06 | 41 | 56.94 |
| Transgression by self/other | 18 | 25.00 | 54 | 75.00 |
| Perceived betrayal | 28 | 38.89 | 44 | 61.11 |
| 9 |  |  |  |  |
| Any PMIE Exposure | 33 | 52.38 | 30 | 47.62 |
| Transgression by self/other | 16 | 25.40 | 47 | 74.60 |
| Perceived betrayal | 29 | 46.03 | 34 | 53.97 |
| 10 |  |  |  |  |
| Any PMIE Exposure | 29 | 52.73 | 26 | 47.27 |
| Transgression by self/other | 20 | 36.36 | 35 | 63.64 |
| Perceived betrayal | 27 | 49.09 | 28 | 50.91 |

*Note.* PMIE = potentially morally injurious event.
