# Supplementary material for: A longitudinal analysis of the role of potentially morally injurious events on COVID-19-related psychosocial functioning among healthcare providers
Source: PLoS One. 2021 Nov 12;16(11):e0260033. doi: 10.1371/journal.pone.0260033 (PMC8589198; doi:10.1371/journal.pone.0260033)
Supplement: S2 Table — (DOCX) [file pone.0260033.s002.docx]

S2 Table.

*Sociodemographic and Work-Related Sample Characteristics based on Reported Exposure to a PMIE*

| Variable | Exposure | | No exposure | |  |
| --- | --- | --- | --- | --- | --- |
|  | *M* | *SD* | *M* | *SD* | *t* |
| Years in profession | 11.25 | 9.14 | 12.39 | 9.49 | .89 |
|  | *n* | % | *n* | % | χ^2^ |
| Sex |  |  |  |  | .73 |
| Male | 15 | 14.29 | 18 | 18.75 |  |
| Female | 90 | 85.71 | 78 | 81.25 |  |
| Race |  |  |  |  | .05 |
| White | 95 | 89.62 | 86 | 88.66 |  |
| Non-White or multiracial | 11 | 10.38 | 11 | 11.34 |  |
| Profession |  |  |  |  | 1.77^a^ |
| Medical provider | 30 | 27.52 | 26 | 25.49 |  |
| Mental health provider | 68 | 62.38 | 61 | 59.80 |  |
| Physical or occupational therapist | 4 | 3.67 | 8 | 7.84 |  |
| Other | 7 | 6.42 | 7 | 6.86 |  |

^a^Fisher’s exact test reported.

*Note.* All comparisons non-significant (*p* > .05). “Medical provider” was comprised of physicians, nurses, nurse practitioners, physician assistants, phlebotomists, EMT/paramedics, and technicians.
