# Supplementary material for: A longitudinal analysis of the role of potentially morally injurious events on COVID-19-related psychosocial functioning among healthcare providers
Source: PLoS One. 2021 Nov 12;16(11):e0260033. doi: 10.1371/journal.pone.0260033 (PMC8589198; doi:10.1371/journal.pone.0260033)
Supplement: S3 Table — (DOCX) [file pone.0260033.s003.docx]

S3 Table.

*Sociodemographic and Work-Related Sample Characteristics based on Reported Exposure to Transgression to Self/Other*

| Variable | Exposure | | No exposure | |  |
| --- | --- | --- | --- | --- | --- |
|  | *M* | *SD* | *M* | *SD* | *t* |
| Years in profession | 10.38 | 8.90 | 12.62 | 9.80 | 1.77 |
|  | *n* | % | *n* | % | χ^2^ |
| Sex |  |  |  |  | 1.70 |
| Male | 9 | 12.00 | 24 | 19.05 |  |
| Female | 66 | 88.00 | 102 | 80.95 |  |
| Race |  |  |  |  | 2.28 |
| White | 71 | 93.42 | 110 | 86.61 |  |
| Non-White or multiracial | 5 | 6.58 | 17 | 13.39 |  |
| Profession |  |  |  |  | 3.32^a^ |
| Medical provider | 19 | 24.68 | 37 | 27.61 |  |
| Mental health provider | 49 | 63.64 | 80 | 59.70 |  |
| Physical or occupational therapist | 2 | 2.60 | 10 | 7.46 |  |
| Other | 7 | 9.09 | 7 | 5.22 |  |

^a^Fisher’s exact test reported.

*Note.* All comparisons non-significant (*p* > .05). “Medical provider” was comprised of physicians, nurses, nurse practitioners, physician assistants, phlebotomists, EMT/paramedics, and technicians.
