# Supplementary material for: A longitudinal analysis of the role of potentially morally injurious events on COVID-19-related psychosocial functioning among healthcare providers
Source: PLoS One. 2021 Nov 12;16(11):e0260033. doi: 10.1371/journal.pone.0260033 (PMC8589198; doi:10.1371/journal.pone.0260033)
Supplement: S4 Table — (DOCX) [file pone.0260033.s004.docx]

S4 Table.

*Sociodemographic and Work-Related Sample Characteristics based on Reported Exposure to Perceived Betrayal*

| Variable | Exposure | | No exposure | |  |
| --- | --- | --- | --- | --- | --- |
|  | *M* | *SD* | *M* | *SD* | *t* |
| Years in profession | 11.90 | 9.10 | 11.73 | 9.49 | -.13 |
|  | *n* | % | *n* | % | χ^2^ |
| Sex |  |  |  |  | 2.14 |
| Male | 10 | 11.90 | 23 | 19.66 |  |
| Female | 74 | 88.10 | 94 | 80.34 |  |
| Race |  |  |  |  | .13 |
| White | 75 | 88.24 | 106 | 89.83 |  |
| Non-White or multiracial | 10 | 11.76 | 12 | 10.17 |  |
| Profession |  |  |  |  | 1.44^a^ |
| Medical provider | 24 | 27.27 | 32 | 26.02 |  |
| Mental health provider | 56 | 63.64 | 73 | 59.35 |  |
| Physical or occupational therapist | 4 | 4.55 | 8 | 6.50 |  |
| Other | 4 | 4.55 | 10 | 8.13 |  |

^a^Fisher’s exact test reported.

*Note.* All comparisons non-significant (*p* > .05). “Medical provider” was comprised of physicians, nurses, nurse practitioners, physician assistants, phlebotomists, EMT/paramedics, and technicians.
